# Potential Biomarkers and Therapeutic Targets in Hepatitis B Virus-related Acute Liver Failure: Interplay of the Ferroptosis, Autophagy and Immune Responses

**DOI:** 10.7150/ijms.106360

**Published:** 2025-01-21

**Authors:** Jing Zuo, Yu-Xin Tian, Qi An, Bai-Yun Wu, Jie-Ru Yang, Yu-Chen Fan

**Affiliations:** 1 Department of Hepatology, Qilu Hospital of Shandong University, Jinan, China.; 2 Institute of Hepatology, Shandong University, Jinan, China.

**Keywords:** hepatitis B virus-related acute liver failure (HBV-ALF), ferroptosis-related, autophagy-related, differentially expressed genes (DEGs)

## Abstract

Hepatitis B virus-related acute liver failure (HBV-ALF) is characterized by a high fatality rate, its pathogenesis remains unclear and the therapeutic efficacy is limited. Ferroptosis which closely related to autophagy may be an underlying mechanism of HBV-ALF. The aim of this study was to identify key ferroptosis- and autophagy-related genes and pathways and provide insight into potential therapeutic approaches for HBV-ALF. We accessed the GSE14668 and GSE96851 datasets from the Gene Expression Omnibus (GEO) database and focused on differentially expressed genes (DEGs), ferroptosis-related DEGs (FRGs) and autophagy-related DEGs (ARGs). Hub genes were subsequently analyzed for enrichment, protein‒protein interactions (PPIs), and different immunological microenvironments, and potential hub gene were identified using MCC method and LASSO. Gene-targeted drugs were from the DGIdb and DrugBank databases.A total of 1462 DEGs were identified (726 upregulated and 736 downregulated). Enriched pathways included amino acid metabolism and immune and inflammatory responses, potentially serving as biomarkers for ALF pathogenesis. After integration with the FerrDb and HADb databases, 55 FRGs and 45 ARGs were identified. Thirteen hub genes (SLC7A11, HMOX1, G6PD, RRM2, KIF20A, HELLS, GPT2, GLS2, SPP1, CCR2, DCN, IRS1, and IGF1) were identified which closely associated with the immune microenvironment. Interplay among these genes occurred primarily through HMOX1. Moreover, we identified several hub gene-targeted drugs that may be effective in HBV-ALF treatment, such as riluzole, acetylcysteine, NADH and Vitamin E.Thirteen hub genes may play crucial roles in HBV-ALF progression, particularly, the HMOX1. Furthermore, drug target exploration offered promising avenues for therapeutic intervention in patients with HBV-ALF.

## Introduction

Acute liver failure (ALF) refers to an acute-onset, life-threatening disease involving multiple systemic manifestations in patients without underlying liver disease and is characterized by severe liver dysfunction and coagulation disorders^1^. Owing to the different etiologies and triggers of ALF worldwide, there is no standard definition of ALF^2^. In developing countries, the main cause of ALF is acute viral hepatitis, which is different from the main cause of ALF in developed countries, where drug-induced liver injury is the predominant cause^2^. The mortality rate of ALF is extremely high, but the pathogenesis has not been fully elucidated, and currently, there are limited treatment prospects. Oxidative stress, systemic inflammatory responses, and immune damage play vital roles in the pathogenesis of ALF. Wang *et al.* reported that glycyrrhizin alleviated ALF damage through the Nrf2/HMOX1/high mobility group box 1 (HMGB1) pathway^1^. Shi *et al.* reported that avicularin can exert a protective effect against ALF by activating the classic Nrf2/HMOX1/glutathione peroxidase 4 (GPX4) antioxidant stress pathway^3^.

The liver plays a crucial role in human metabolism, including that of the three major nutrients and iron^4^. Iron overload can lead to the occurrence of liver ferroptosis^5^, ^6^. Ferroptosis is an iron-dependent form of regulated cell death characterized by lipid peroxidation and iron metabolism disorders and is closely related to oxidative stress damage in the body^7^. Moreover, ferroptosis plays an important role in the pathogenesis of liver diseases, with studies showing that acetaminophen (APAP)-induced ALF is related to ferroptosis^8^. Ferroptosis also promotes liver ischemia‒reperfusion injury after liver transplantation, and plays a role in the pathogenesis of hemochromatosis, autoimmune liver disease, nonalcoholic fatty liver disease, hepatocellular carcinoma and other liver diseases^9^-^14^. Glutathione (GSH)/GPX4 can alleviate oxidative stress damage, and subunit solute carrier family 7 member 11 (SLC7A11) is a key regulatory factor of ferroptosis that can transport cysteine into cells and reduce it to cysteine, providing raw materials for GSH^15^, ^16^. Autophagy is a highly conserved lysosomal degradation pathway. Unlike cellular autophagy and cuproptosis, ferroptosis is an autophagy-dependent form of cell death, and GPX4 and SLC7A11 are also important regulatory factors of autophagy^17^, ^18^.

The mechanisms of ferroptosis and autophagy in HBV-ALF are not yet fully understood. However, inhibiting ferroptosis in hepatocytes can alleviate liver damage, suggesting that ferroptosis might represent a potential therapeutic target for HBV-ALF. To explore this, bioinformatics was utilized to screen for differentially expressed genes (DEGs) related to ferroptosis and autophagy in ALF. Gene enrichment analysis and immunoenvironmental analysis were subsequently conducted to identify hub genes and investigate their potential as biomarkers and the differences in the immunological microenvironment between groups, thus providing theoretical support for the development of subsequent diagnostic and treatment strategies. Our research findings suggested that SLC7A11, HMOX1, G6PD, RRM2, KIF20A, HELLS, GPT2, GLS2, SPP1, CCR2, DCN, IRS1, and IGF1 may play significant roles in HBV-ALF, and there were notable changes in the immune microenvironment in HBV-ALF.

## Materials and Methods

### Screening of microarray data

Data for patients with ALF were obtained from the NCBI Gene Expression Omnibus (GEO, https://www.ncbi.nlm.nih.gov/). We downloaded the GSE14668 and GSE96851 datasets, which were derived from the GPL570 platform (Affymetrix Human Genome U133 Plus 2.0 Array)^19^, ^20^. Employing dual datasets, transcriptomic analysis was conducted using hepatic resection samples. The study cohort included individuals with ALF (n=6), individuals who underwent surgical resection for angioma (n=27), and healthy individuals (HCs) (n=9). The data for 25 liver tissue samples from ALF patients and 37 healthy liver tissue samples were selected for advanced analysis.

### Identification of ferroptosis- and autophagy-related DEGs

R (4.4.1) and “Bioconductor”packages were used to analyze the significance of the DEGs between the HBV-ALF samples and healthy liver samples. The R programming environment and Bioconductor project provide a rich collection of tools specifically designed for the analysis of genomic data, enabling robust statistical testing and comprehensive bioinformatics workflows. The GSE14668 and GSE96851 datasets were normalized. The DEG identification threshold values were set at |log2-fold change (FC)|≥ 1.5 and P < 0.05. We subsequently downloaded 564 genes from the Ferroptosis Database (FerrDb V2) (http://www.zhounan.org/ferrdb/current/) and compared those genes with the GSE14668 and GSE96851 datasets to screen for ferroptosis-related DEGs (FRGs)^21^. Similarly, we retrieved 794 genes from the HADb (http://www.autophagy.lu) and intersected those genes with those in the GSE14668 and GSE96851 datasets. The R software packages “VennDiagram” and “ggplots” were used to plot Venn diagrams and volcano maps.

### Functional enrichment analysis

Functional enrichment analysis of the DEGs was performed using the “msigdbr”, “org.Hs.eg.db”, and “clusterProfiler” packages in R. Default parameters were almost utilized. The gene set references were derived from the MSigDB collections. After the DEGs were uploaded, ID conversion of the input data was conducted. The “clusterProfiler” package was subsequently employed to carry out gene set enrichment analysis (GSEA) to explore the biological functions of the DEGs. Kyoto Encyclopedia of Genes and Genomes (KEGG) analysis was performed using the GSEA results. Using R, we performed KEGG pathway enrichment analysis to identify the intrinsic pathways associated with the target genes. In addition, we performed Gene Ontology (GO) categorization for and KEGG pathway enrichment analysis of DEGs involved in ferroptosis and autophagy, employing the "enrichKEGG" and "enrichGO" functions with the "clusterProfiler" package. Moreover, we identified FRGs across biological processes (BPs), cellular components (CCs), and molecular functions (MFs). KEGG pathway enrichment analysis was subsequently conducted. To visualize the enrichment outcomes, we used the "ggplot2" package.

### Establishment of a PPI network and identification of hub genes

Protein‒protein interaction (PPI) networks of ferroptosis-related and autophagy-related DEGs were constructed using STRING (version 12.0, https://cn.string-db.org/). Graphical representations were generated using Cytoscape (version 3.9.0, https://cytoscape.org/). We applied the Molecular Complex Detection (MCODE) algorithm in Cytoscape to conduct a cluster analysis of the PPI network using the following parameters: degree cutoff, 2; node score threshold, 0.2; kcore value, 2; and maximum depth, 100. Furthermore, CytoHubba was used to identify the top 10 pivotal nodes, and an intersection-based approach was used to refine the selection of hub genes. For this process, the maximum clique centrality (MCC) method was used. In the Stata 17, to reduce the data dimensionality, the least absolute shrinkage and selection operator (LASSO) algorithm was used to identify gene biomarkers for HBV-ALF. The LASSO analysis results were intersected with the MCC results to select hub genes. Besides, the construction of the network graph was facilitated using the “igraph” and “ggraph” packages.

### Immune cell infiltration analysis

We imported the expression profile data into CIBERSORTx (https://cibersortx.stanford.edu/) to assess the immune landscape^22^. We predicted the proportions of 22 infiltrating immune cell types in each tissue. We subsequently utilized the ggplot2 package in R to visualize the data.

### Drugs identified in the DGIdb and DrugBank

Drugs targeting the identified hub genes were sourced from the Drug-Gene Interaction Database (DGIdb) (https://www.dgidb.org/) and DrugBank (https://go.drugbank.com/) databases^23^, ^24^. Both databases provide detailed information on drugs and their corresponding targets, allowing for the investigation of the pharmacological effects of these drugs as well as their current status in the drug development pipeline.

### Statistical analysis

R version 4.4.1 and Stata 17 were used for the statistical analyses and for generating graphs. Student's t test was used to assess significant differences in means between two groups. The thresholds for statistical significance were *P < 0.05, **P < 0.01, and ***P < 0.001.

## Results

### DEGs in HBV-ALF and enrichment analysis

**Figure 1:**
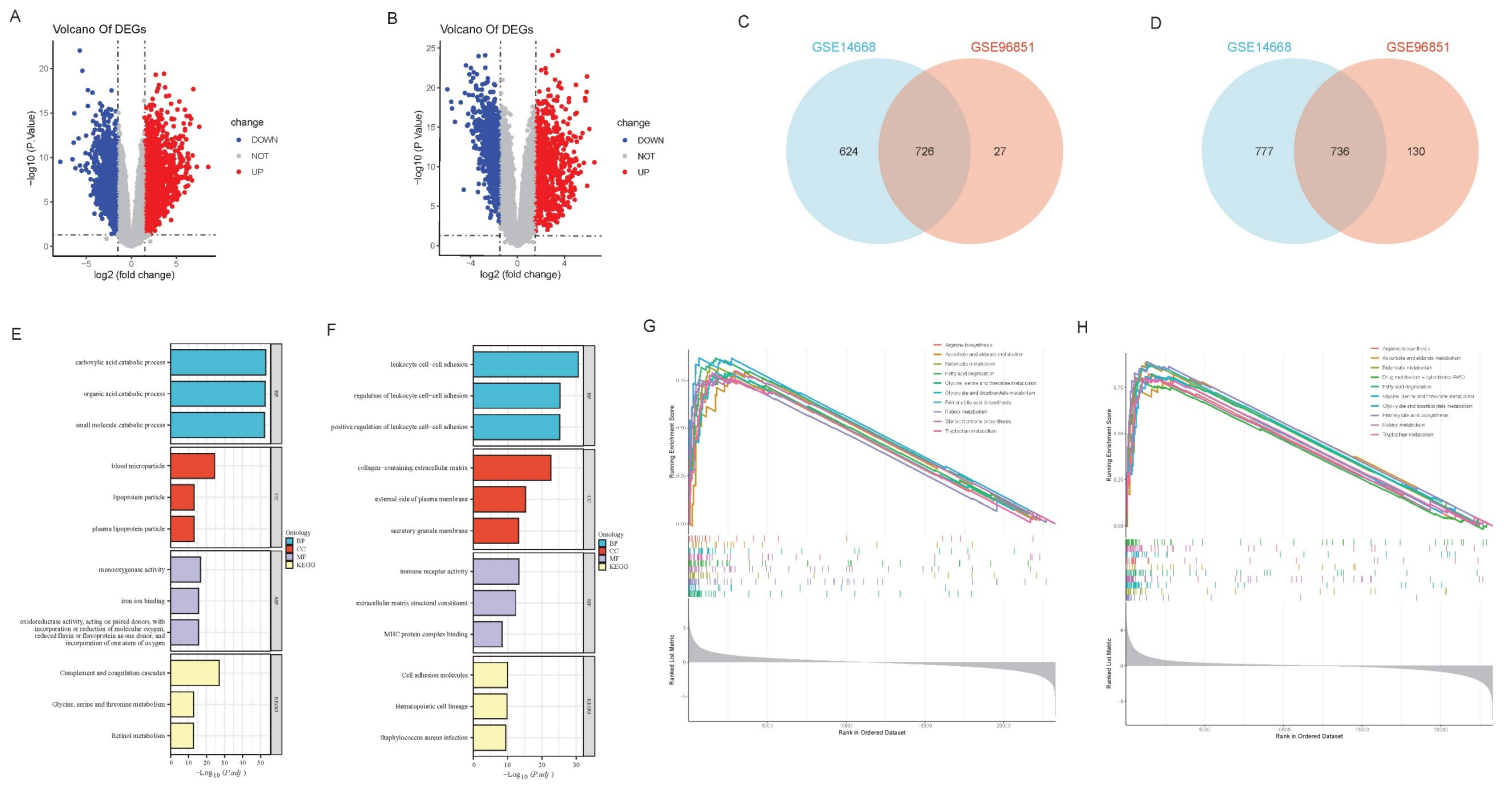
** DEGs in HBV-ALF and enrichment analysis.** (A) Volcano plot of differentially expressed genes (DEGs) between the HBV-ALF group and control group, derived from the GSE14668 dataset, with red representing significantly upregulated genes and blue representing significantly downregulated genes. Two vertical lines indicate a fold change (FC) > 2 and < - 2, respectively, in gene expression, and the horizontal line indicates the adjusted P value (FDR q-value) of 0.05. P values were calculated by two-sided Wilcoxon rank-sum test. The color of the dot represents the FDR (q-value) levels (B) Volcano plot of DEGs in the GSE96851 dataset (C) Venn diagram of the common upregulated DEGs in GSE14668 and GSE96851 (D) Venn diagram of the common downregulated DEGs in GSE14668 and GSE96851 (E) Gene Ontology (GO) enrichment analysis and Kyoto Encyclopedia of Genes and Genomes (KEGG) pathway analysis of upregulated DEG (F) GO enrichment analysis and KEGG pathway analysis of downregulated DEGs (G) Gene set enrichment analysis (GSEA) revealed the pathways enriched with upregulated DEGs (H) GSEA revealed the pathways enriched with the downregulated DEGs.

To determine whether ferroptosis and autophagy play pivotal roles in HBV-ALF, we initially analyzed the biological functions and enriched pathways of the common DEGs across two datasets. The GSE14668 and GSE96851 datasets, which included 25 liver tissue samples from ALF patients and 37 healthy liver tissue samples, were obtained from the GEO database. A total of 1350 upregulated DEGs and 1513 downregulated DEGs were identified in the GSE14668 dataset, and 753 upregulated DEGs and 866 downregulated DEGs were identified in the GSE96851 dataset (Fig. 1A, B); in total, 726 upregulated DEGs and 736 downregulated DEGs were identified in both datasets (Fig. 1C, D). The MFs of the upregulated genes were predominantly associated with iron ion metabolism and oxidative stress-related functions. KEGG enrichment analysis revealed that the genes were enriched in pathways associated with complement and coagulation functions, as well as glycine, serine, and threonine metabolism and retinol metabolism (Fig. 1E). The MFs of the downregulated genes were associated mainly with immune-related functions and transplant rejection, with pathway enrichment indicating associations with cell adhesion molecules, hematopoiesis functions, and Staphylococcus aureus infection (Fig. 1F). GSEA of the two datasets revealed that the differentially expressed genes were enriched primarily in molecular metabolic pathways (Fig. 1G, H). As demonstrated in the results, the MFs of a portion of DEGs are correlated with iron metabolism, indicating that ferroptosis and autophagy may be one of the significant pathogenic mechanisms in HBV-ALF. Based on these observations, we proceeded to the subsequent analysis.

### FRGs, hub genes of FRGs and enrichment analysis

**Figure 2:**
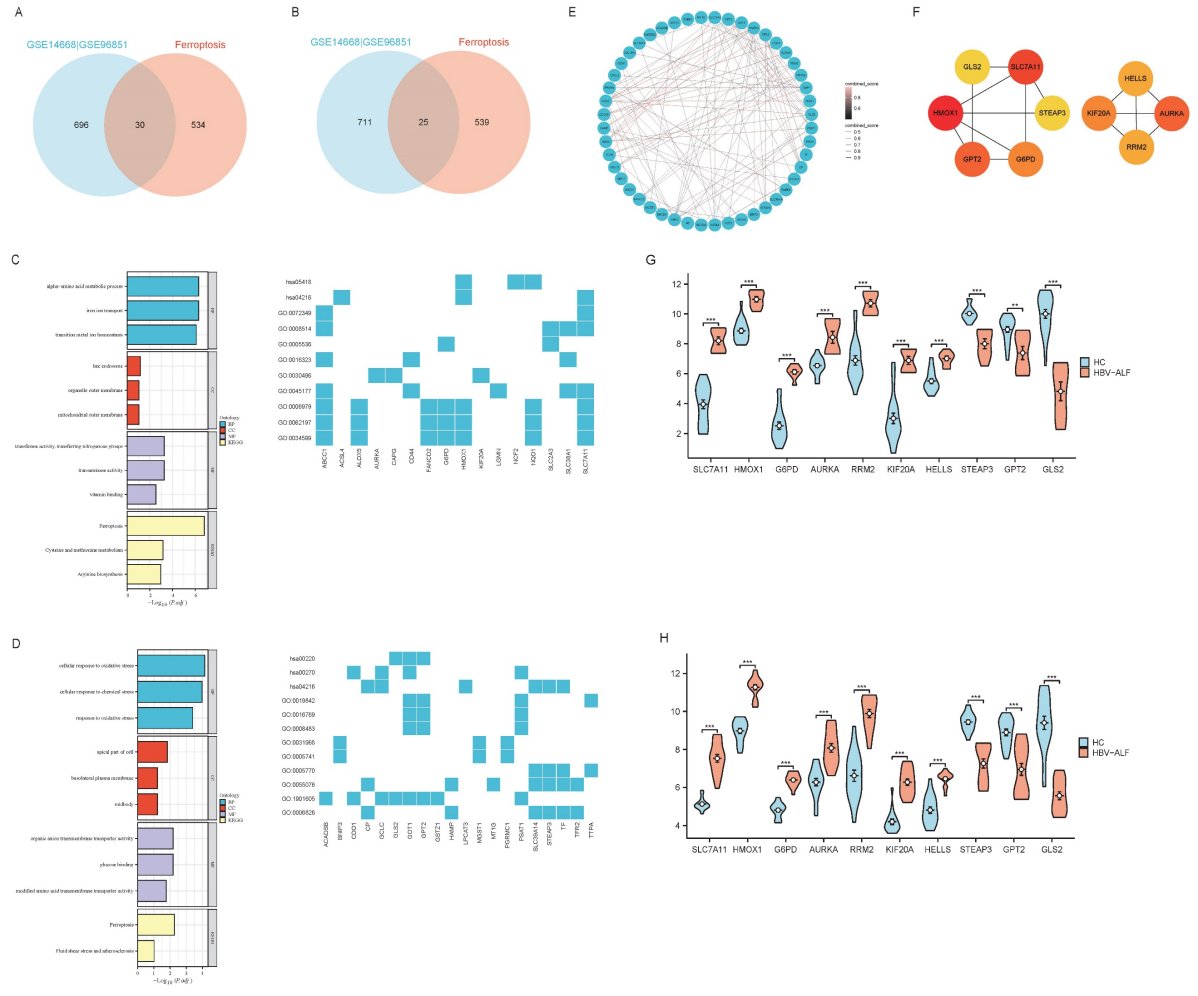
** FRGs, hub genes of FRGs and enrichment analysis.** (A) The R software packages “VennDiagram” were used to plot Venn diagrams of the common upregulated DEGs among GSE14668, GSE96851 and ferroptosis-related genes. The construction of the network graph was facilitated using the “igraph” and “ggraph” packages (B) Venn diagram of the common downregulated DEGs among GSE14668, GSE96851 and ferroptosis-related genes (C) GO enrichment analysis and KEGG pathway analysis of upregulated ferroptosis-related DEGs (FRGs) (D) GO enrichment analysis and KEGG pathway analysis of downregulated FRGs (E) A network graph of the 55 FRGs (F) In Cytoscape, the maximum connectivity clustering (MCC) algorithm was used to identify the top 10 highly connected ferroptosis-related genes (G) Expression levels of the top ten ferroptosis-related gene biomarkers between the patients with HBV-ALF and normal controls in GSE14668 (H) Expression levels of the top ten ferroptosis-related gene biomarkers for patients with HBV-ALF and normal controls in GSE96851.

In order to further analyze the role and mechanism of ferroptosis and its associated genes in HBV-ALF, we obtained 564 genes from the FerrDb database and then compared them with genes in the GSE14668 and GSE96851 datasets to identify FRGs. Accordingly, fifty-five genes were identified, with thirty being upregulated and twenty-five being downregulated (Fig. 2A, B and E). The FRGs were further categorized into driver, suppressor, marker and unclassified genes using the online tool FerrDb online which was shown in Table 1. The upregulated genes were associated primarily with iron ion transport and the metabolism of amino acids such as alanine, aspartate, and glutamate, and the downregulated genes were closely related to oxidative stress biological processes and pathways involved in fluid shear stress and atherosclerosis (Fig. 2C, D). The following ten genes were screened using the MCC method in CytoHubba within Cytoscape: SLC7A11, HMOX1, G6PD, AURKA, RRM2, KIF20A, HELLS, STEAP3, GPT2, and GLS2 (Fig. 2F). The expression of these 10 genes was significantly different between the healthy control group and the HBV-ALF group. Specifically, in the HBV-ALF group, SLC7A11, HMOX1, G6PD, AURKA, RRM2, KIF20A and HELLS expression was increased, and STEAP3, GPT2, and GLS2 expression was decreased (Fig. 2G,H). Through variable selection with LASSO and intersection with the 10 genes, 8 genes were identified: SLC7A11, HMOX1, G6PD, RRM2, KIF20A, HELLS, GPT2 and GLS2 (Fig. S1).

### ARGs, hub genes of ARGs and enrichment analysis

**Figure 3:**
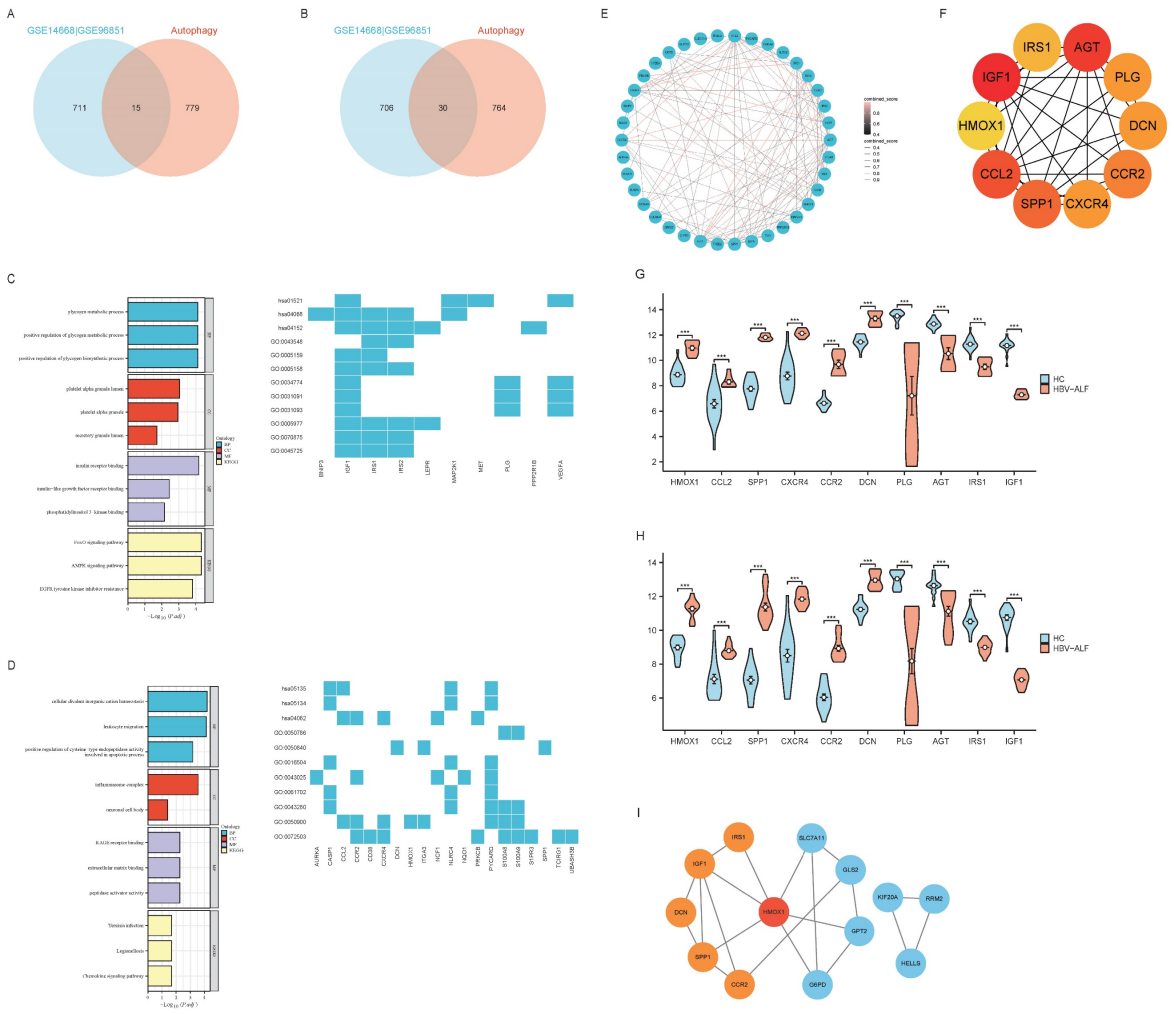
** ARGs, hub genes of ARGs and enrichment analysis.** (A) The R software packages “VennDiagram” were used to plot Venn diagrams of the common upregulated DEGs among GSE14668, GSE96851 and autophagy-related genes. The construction of the network graph was facilitated using the “igraph” and “ggraph” packages (B) Venn diagram of the common downregulated DEGs among GSE14668, GSE96851 and autophagy-related genes (C) GO enrichment analysis and KEGG pathway analysis of upregulated autophagy-related DEGs(ARGs) (D) GO enrichment analysis and KEGG pathway analysis of downregulated ARGs; E A network graph of the 45 ARGs (F) In Cytoscape, the maximum connectivity clustering (MCC) algorithm was used to identify the top ten highly connected autophagy-related genes (G) Expression levels of the top ten autophagy-related gene biomarkers for patients with HBV-ALF and normal controls in GSE14668 (H) Expression levels of the top ten autophagy-related gene biomarkers for patients with HBV-ALF and normal controls in GSE96851 (I) The association network graph for ferroptosis-related and autophagy-related hub genes.

Ferroptosis is an autophagy-dependent form of cell death. Therefore, in this section, we analyzed the role of ARGs in HBV-ALF. A total of 794 genes were obtained from HADb and then compared with genes in the GSE14668 and GSE96851 datasets to identify ARGs. Forty-five genes were identified, with fifteen being upregulated and thirty being downregulated (Fig. 3A, B and E). The upregulated genes were enriched in pathways associated with the cell cycle, cell proliferation, antioxidant stress, and energy metabolism. Moreover, the downregulated genes were involved primarily in infection, immune defense, and chemical signaling pathways (Fig. 3C, D). Ten genes were selected using the MCC method: HMOX1, CCL2, SPP1, CXCR4, CCR2, DCN, PLG, AGT, IRS1, and IGF1 (Fig. 3F). The expression of these genes was significantly different between the two groups. Specifically, in the HBV-ALF group, the expression of HMOX1, IGF1, CCL2, SPP1, CCR2, and CXCR4 was upregulated, and the expression of AGT, PLG, DCN, and IRS1 was downregulated (Fig. 3G-H). By intersecting the LASSO results with the MCC results, the hub genes HMOX1, SPP1, CCR2, DCN, IRS1, and IGF1 were identified (Fig. S2). The identified hub genes interact in ferroptosis and autophagy processes through HMOX1, which plays a vital role in both ferroptosis and autophagy (Fig. 3I).

### Immune infiltration and correlation analysis

**Figure 4:**
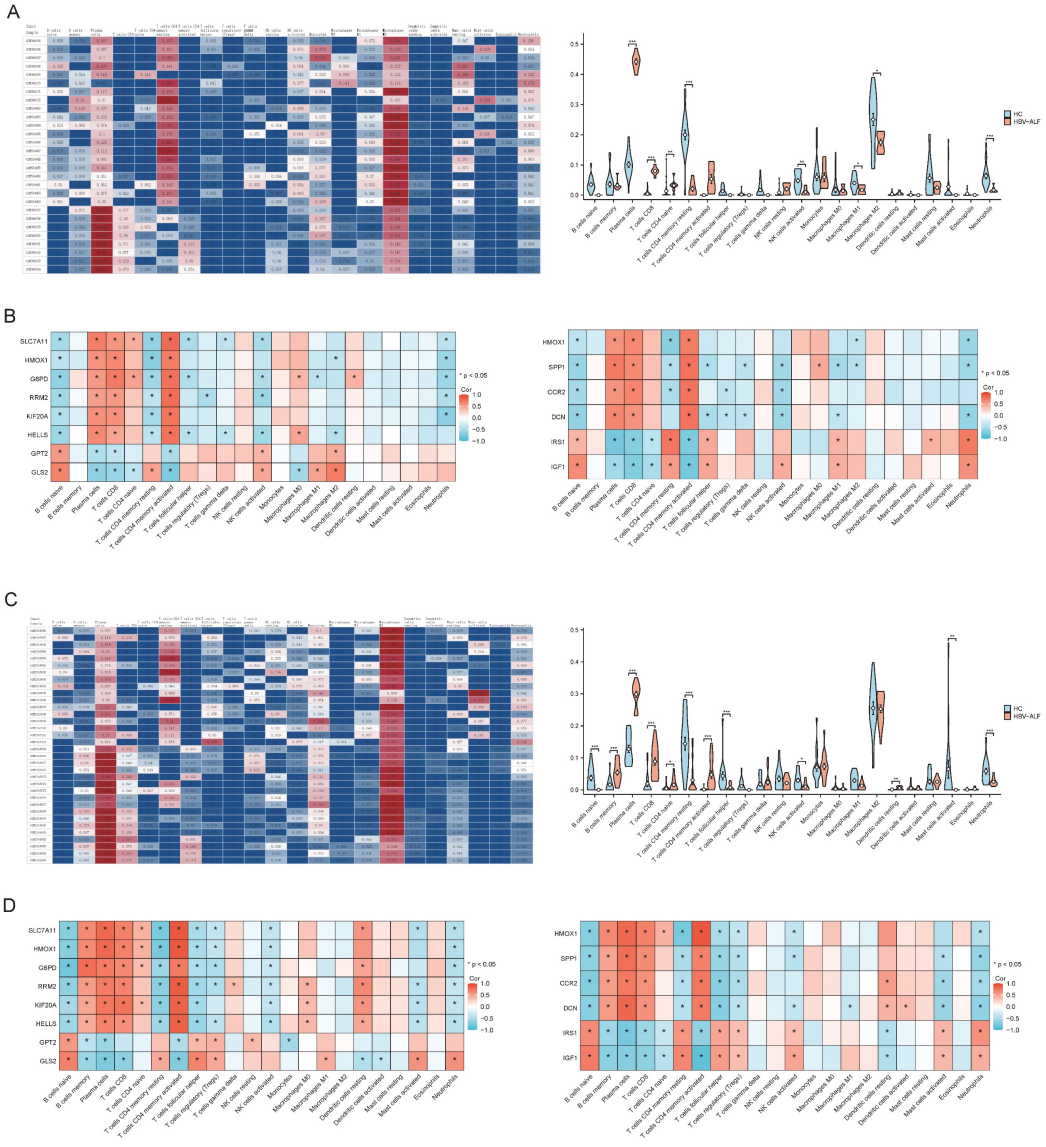
** Immune infiltration and correlation analysis.** Characteristics of infiltrating immune cells. Proportions of 22 immune cell subpopulations in liver tissues. Correlation coefficient heatmap to visualize the interactions among hub genes and immune cells. (A) GSE14668 (C) GSE96851. Violin plot showing the immune cells with differential infiltration (*p < 0.05) (B) GSE14668 (D) GSE96851.

Gene enrichment analysis suggested that the DEGs were closely related to immune functions. We conducted immune cell infiltration analysis of the GSE14668 and GSE96851 datasets using CIBERSORTx to further differentiate the distinct immunological microenvironments between the HBV-ALF and HC groups. In both datasets, the proportions of plasma cells, CD8+ T cells, and naive CD4+ T cells in patients with HBV-ALF were significantly greater than those in HCs. In contrast, the proportions of CD4 memory resting T cells, activated NK cells, and neutrophils were significantly lower in patients with HBV-ALF than in HCs (Fig. 4A, C and Table S1, 2). Correlation analysis revealed that SLC7A11, HMOX1, G6PD, RRM2, KIF20A, HELLS, SPP1, CCR2 and DCN were positively correlated with plasma cells and CD8+ T cells, whereas GPT2, GLS2, IRF1, and IGF1 were negatively correlated with these cell subsets (Fig. 4B, D). The opposite trends were observed for neutrophils (Fig. 4B, D). For both datasets, SLC7A11 was positively correlated with CD4 naive T cells and negatively correlated with activated NK cells and neutrophils (Fig. 4B, D). What's more, HMOX1 was negatively correlated with T cells CD4 memory resting and neutrophils (Fig. 4B, D). The aforementioned findings suggested that SLC7A11 and HMOX1 may exert their effects by modulating the quantity of relevant innate or adaptive immune cells, as well as their recruitment and immune functions.

### Common DEGs (co-DEGs)

**Figure 5:**
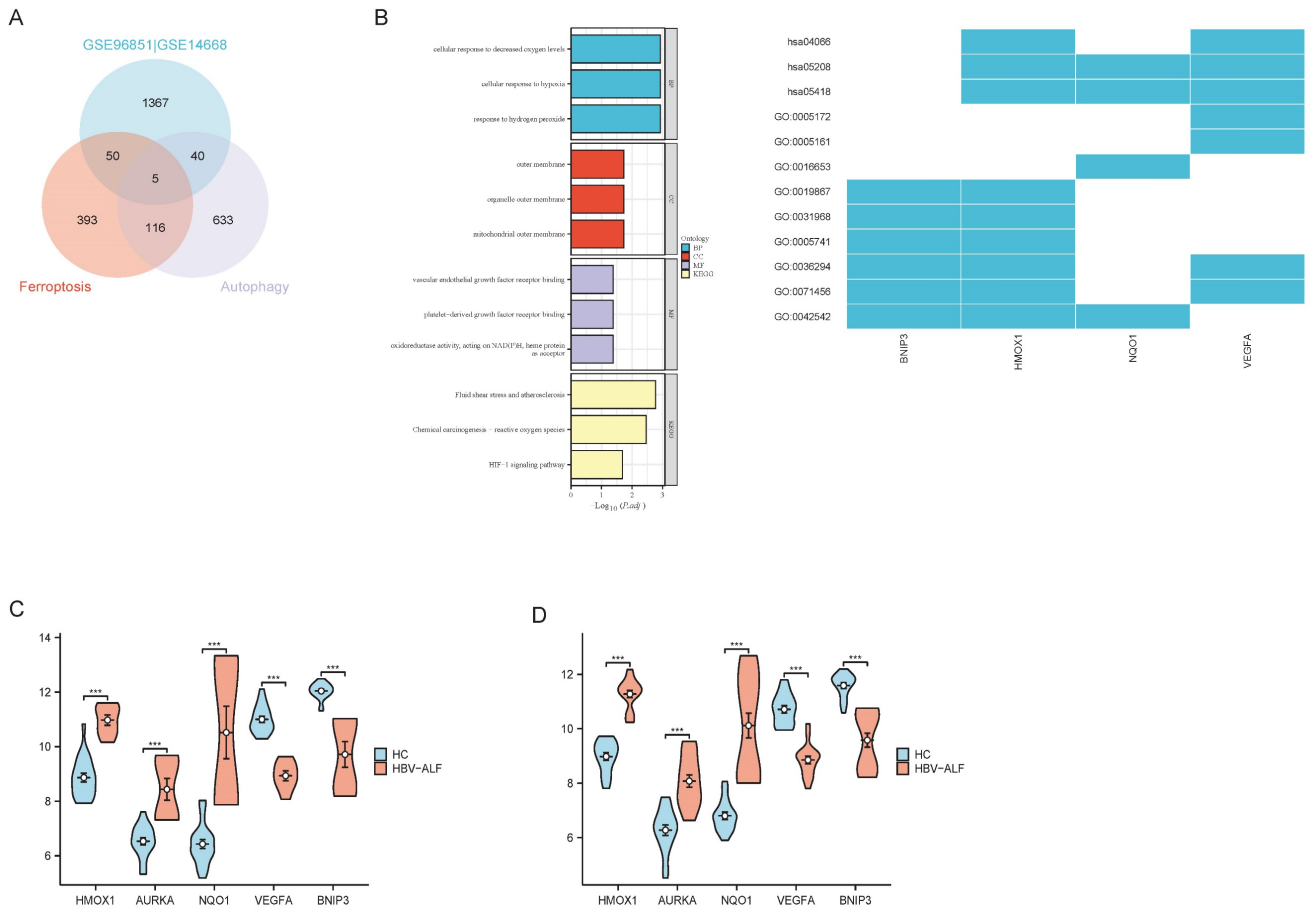
** Common DEGs (co-DEGs).** The R package 'VennDiagram' was used to plot Venn diagrams. (A) Venn diagrams of the common DEGs among the two datasets and ferroptosis-related and autophagy-related genes (B) GO enrichment analysis and KEGG pathway analysis of co-DEGs (C) Expression levels of the five co-DEGs in GSE14668 (D) Expression levels of the five co-DEGs in GSE96851.

The abovementioned results suggest that in patients with HBV-ALF, DEGs interact in ferroptosis and autophagy processes. We identified five co-DEGs among the two datasets that were associated with both ferroptosis and autophagy DEGs. Among these genes, the expression of HMOX1, AURKA, and NQO1 was upregulated, whereas the expression of VEGFA and BNIP3 was downregulated (Fig. 5A, C and D). Gene enrichment analysis revealed that the five genes were enriched primarily in oxidative and antioxidant signaling pathways (Fig. 5B) and that the expression of these genes was significantly different between the HBV-ALF and HC groups (Fig. 5C, D).

### Ferroptosis-related hub gene-targeted drugs

**Figure 6:**
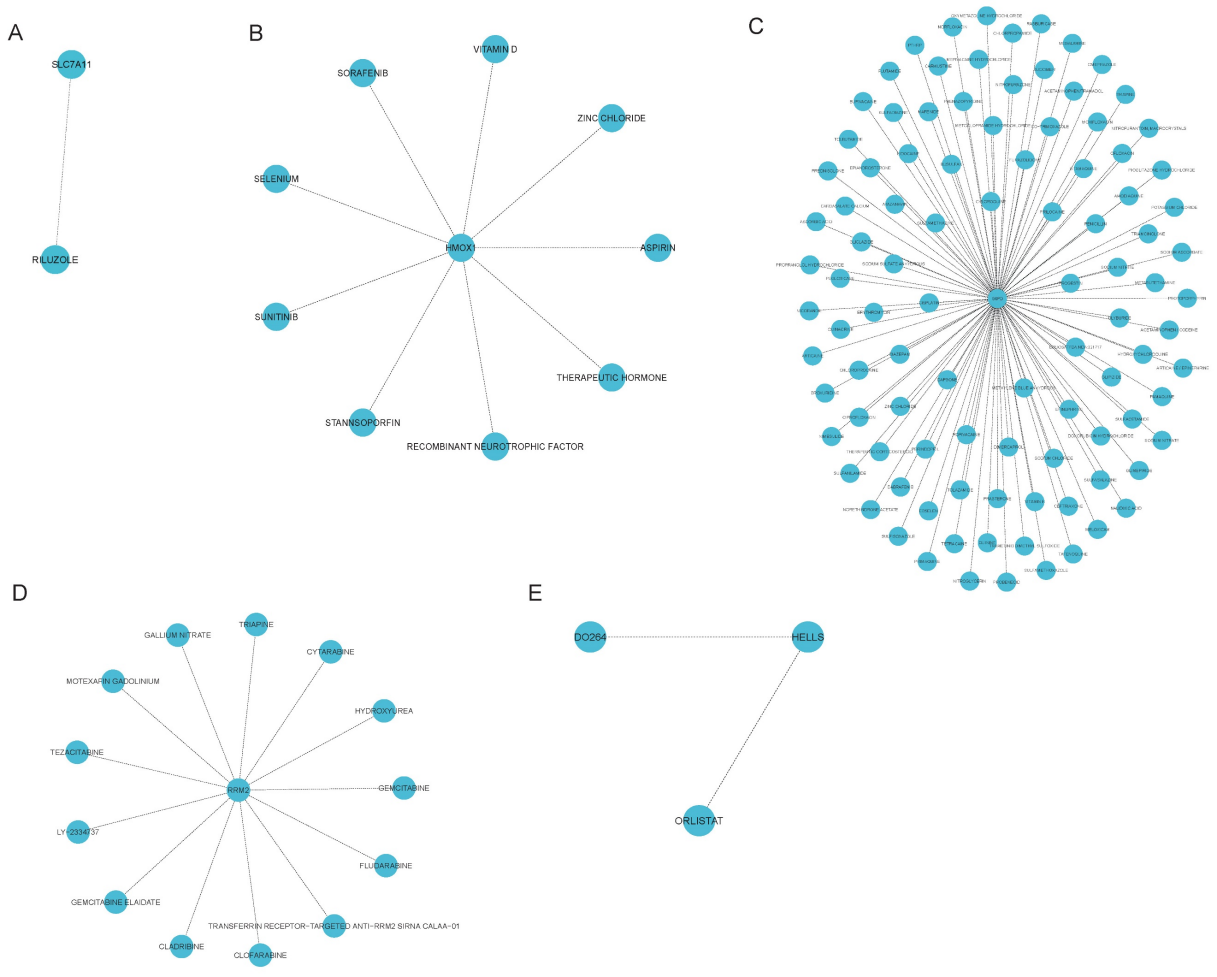
** Ferroptosis-related hub gene-targeted drugs.** Network graphs were constructed using the R packages 'igraph' and 'ggraph'. An association network graph of ferroptosis-related hub gene-targeted drugs. (A) SLC7A11-targeted drugs (B) HMOX1-targeted drugs (C) G6PD-targeted drugs (D) RRM2-targeted drugs (E) HELLS-targeted drugs.

We utilized the DGIdb to identify ferroptosis-related gene-targeted drugs. In total, we discovered 125 drugs that targeted marker genes, including 1 for SLC7A11, 9 for HMOX1, 100 for G6PD, 13 for RRM2 and 2 for HELLS (Fig. 6A-E). Unfortunately, we did not find targeted drugs for KIF20A, GPT2 and GLS2. We subsequently searched DrugBank for approved gene-targeted drugs, yielding 8 for SLC7A11, 2 for HMOX1, 2 for G6PD, 2 for RRM2, 3 for GPT2. However, drugs targeting the other three genes were not identified. Taking SLC7A11-targeted drugs as an example, riluzole, which has been shown to affect glutamate uptake and transport in neurological disorders, was commonly identified in both databases^25^, ^26^. Aspirin, zinc chloride, vitamin D, sorafenib, selenium, sunitinib, and stannsoporfin were identified as HMOX1-targeted drugs in the DGIdb dataset, whereas NADH (DB00157) and vitamin E (DB00163) were found in the DrugBank dataset which was shown in Table 2. These drugs are associated primarily with antioxidant stress responses. Glycolic acid (DB03085), artenimol (DB11638) and prasterone (DB01708) are G6PD-targeted drugs. Cladribine (DB00242) and Gallium nitrate (DB05260) are RRM2-targeted drugs. Both gene-targeted drugs were shown in Table 2. Furthermore, despite the absence of GPT2-targeted drugs in the DGIdb, we identified pyridoxal phosphate (DB00114), glutamic acid (DB00142), and phenelzine (DB00780) in DrugBank in Table 2.

### Autophagy-related hub gene-targeted drugs

**Figure 7:**
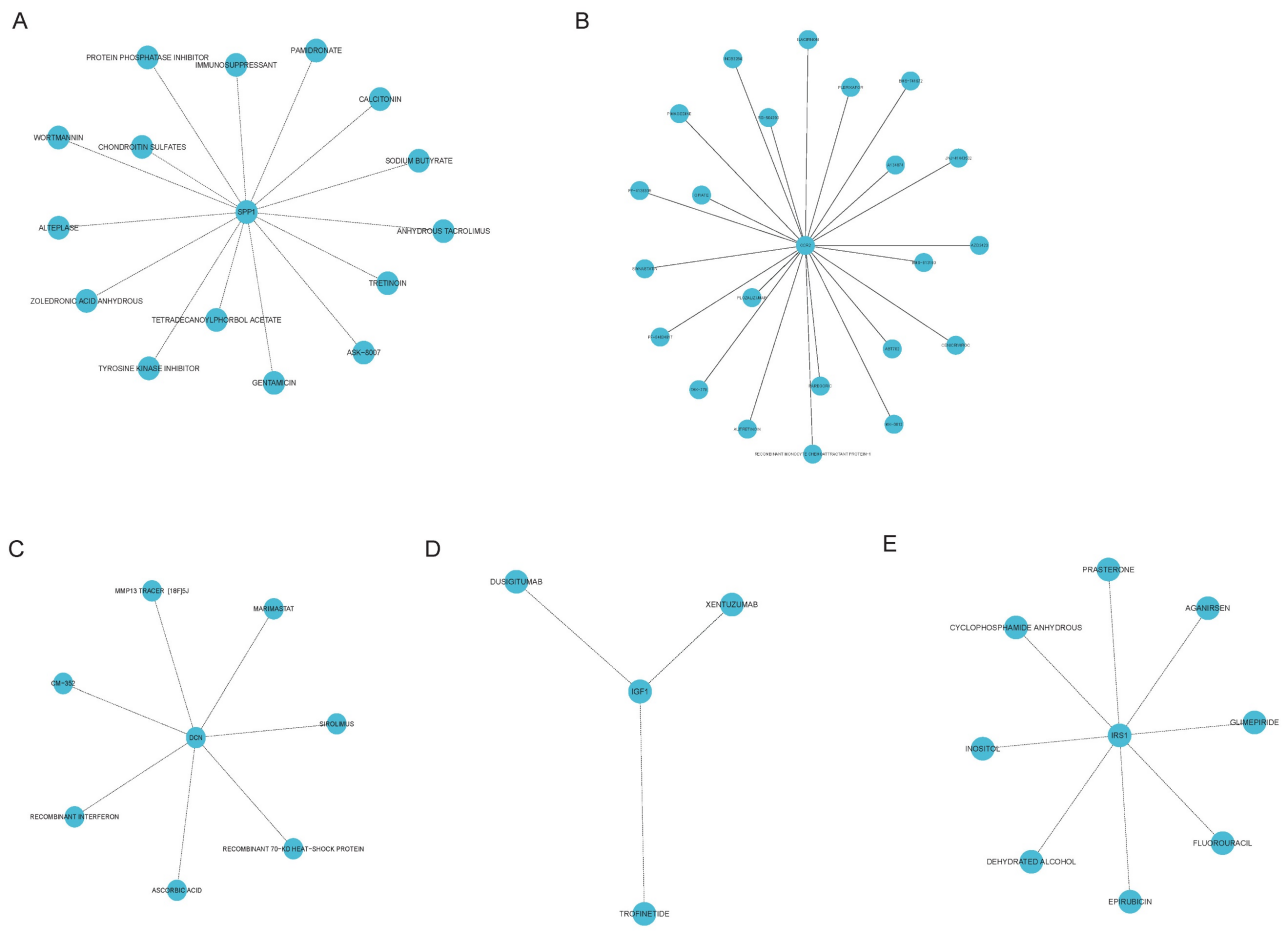
** Autophagy-related hub gene-targeted drugs.** Network graphs were constructed using the R packages 'igraph' and 'ggraph'. An association network graph of autophagy-related hub gene-targeted drugs. (A) SPP1-targeted drugs (B) CCR2-targeted drugs (C) DCN-targeted drugs (D) IGF1-targeted drugs (E) IRS1-targeted drugs.

We also searched the DGIdb for autophagy-related gene-targeted drugs. We identified 56 drugs that targeted marker genes, including 9 for HMOX1, 15 for SPP1, 23 for CCR2, 7 for DCN, 8 for IRS1, and 4 for IGF1 (Fig. 6B and Fig. 7A-E). We subsequently queried the DrugBank database for approved gene-targeted drugs and identified 2 drugs that target HMOX1 which was shown in Table 3. However, no targeted drugs were found for the other 5 genes.

## Discussion

HBV-ALF is a liver disease with a high mortality rate. Previous studies have indicated that various modes of cell death played significant roles in liver diseases, yet their mechanisms in the pathogenesis of HBV-ALF remained unclear. Oxidative stress, systemic inflammatory responses, and immune damage are crucial in the pathogenesis of ALF. Ferroptosis, characterized by dysregulated iron metabolism and lipid peroxidation, is an autophagy-dependent form of cell death that plays a vital role in oxidative stress. Therefore, our study analyzed the role of ferroptosis- and autophagy-related DEGs in HBV-ALF, with the aim of identifying potential therapeutic targets. SLC7A11, HMOX1, G6PD, RRM2, KIF20A, HELLS, GPT2, GLS2, SPP1, CCR2, DCN, IRS1, and IGF1 may play vital roles in HBV-ALF progression. Certain drugs targeting hub genes such as Riluzole, acetylcysteine, vitamin E and NADH, may hold therapeutic promise in the treatment of HBV-ALF.

In our study, 726 upregulated DEGs and 736 downregulated DEGs were identified in the GSE14668 and GSE96851 datasets. We identified the hub genes associated with ferroptosis and autophagy and explored their roles in the onset or treatment of HBV-ALF. In summary, fifty-five common DEGs, consisting of thirty upregulated genes and twenty-five downregulated genes, were selected from both datasets and the FerrDb dataset. GO enrichment analysis revealed that the ferroptosis-related upregulated DEGs were associated primarily with iron ion transport and the metabolism of amino acids, including alanine, aspartate, and glutamate, whereas the downregulated genes were closely associated with oxidative stress-related biological processes and pathways such as fluid shear stress and atherosclerosis. Eight hub genes were identified, namely, SLC7A11, HMOX1, G6PD, RRM2, KIF20A, HELLS, GPT2 and GLS2. The cross-comparison of DEGs from the two datasets and HADb yielded 45 genes. Fifteen DEGs were upregulated, and 30 DEGs were downregulated. GO enrichment analysis suggested that the autophagy-related upregulated DEGs were enriched in pathways closely related to the cell cycle, cell proliferation, antioxidant stress, and energy metabolism functions, whereas the downregulated genes were enriched in pathways related to infection, immune defense, and chemical signaling. The hub genes were HMOX1, SPP1, CCR2, DCN, IRS1, and IGF1. HMOX1 was a common gene.

Ferroptosis is a form of regulated cell death that is dependent on autophagy, and there are regulatory factors that are shared between the two processes. Our results revealed that ferroptosis-related DEGs connected with autophagy-related DEGs through HMOX1, further suggesting a close association between ferroptosis and autophagy^18^, ^27^. Lei *et al.* reported that Med1 might inhibit ferroptosis by activating the Nrf2/HMOX1 pathway, thereby alleviating liver injury in ALF^28^. The GSH/GPX4 pathway is a classic pathway for inhibiting ferroptosis. Glutamate, cysteine, and glycine are precursors for GSH, and GPX4 inactivation can induce ferroptosis via GSH/GPX4^29^. Cysteine is the limiting amino acid in GSH synthesis, and SLC7A11 assists in GSH synthesis, using cysteine as a substrate, and plays a role in the antioxidant stress response. The overexpression of SLC7A11 inhibits ferroptosis^30^. Studies have suggested that SLC7A11 is also a regulator of autophagy; however, we did not find this gene in the HADb database^31^, ^32^. G6PD deficiency is a common inherited disorder characterized by hemolysis and has been suggested to be associated with the onset of ALF in case reports^33^. However, the role of ferroptosis in HBV-ALF still needs to be investigated, and our results suggest that genes related to ferroptosis and autophagy play roles in the pathogenesis of HBV-ALF.

Our immune cell infiltration analysis revealed differences in the immunological microenvironment between patients with HBV-ALF and healthy controls. Omata and Liang *et al.* have demonstrated that mutations in the precore region of the hepatitis B virus (HBV) genome affected the coding of HBV e antigen (HBeAg), which was associated with severe immunological dysregulation in acute severe hepatitis^34^, ^35^. Specifically, these studies identified a G-to-A mutation at nucleotide positions 1896 or 1898, converting tryptophan (TGG) to a stop codon (TAG), leading to the absence of HBeAg^34^, ^35^. This absence may result in upregulation of HBV core antigen expression, intensifying the host's immune response or altering the antigenic characteristics of HBV, thereby causing significant immunological damage. Additionally, Sato *et al.* found that HBV strains associated with liver damage may not present mutations in the precore region but rather in the core promoter, affecting HBeAg transcription^36^. It is also observed that HBeAg is often negative in patients with acute severe hepatitis. What's more, exposure to body fluids from patients with low viral loads or positive for HBeAb may precipitate the onset of acute severe hepatitis, which warranted attention. Beyond mutations in the HBeAg-coding domain, Chen *et al.* have shown that, in patients with HBV-ALF, there is a significant accumulation of plasma cells in the necrotic areas of the liver and that the HBV core antigen (HBcAg) is highly mutated. Therefore, plasma cells produce large amounts of IgM and IgG antibodies that target HBcAg. The formation of antigen‒antibody complexes activates the complement system, leading to massive hepatocyte death, suggesting that HBV-ALF may be an HBV core-driven B cell disease^19^. The baseline characteristics of the enrolled patients in the two datasets we analyzed showed that nearly all were HBeAg-negative and HBeAb-positive, consistent with previous research findings. Our results revealed that compared with that of other cell subsets, the number of plasma cells was greater in the HBV-ALF group, indicating the important role of B cells in the pathogenesis of HBV-ALF. In addition, the proportions of CD8+ T cells and naive CD4+ T cells in patients with HBV-ALF significantly increased. T cell-related subtypes may become functionally dysregulated or exhausted during acute HBV infection, CHB, and the development of HBV-ALF and may serve as important therapeutic targets, suggesting that they may also cause certain numerical or functional disorders in patients with HBV-ALF^37^-^39^.

Innate immune cells play a significant role in the pathogenesis of HBV-ALF by influencing the adaptive immune response and secreting antiviral cytokines, including interferon-γ and tumor necrosis factor-alpha. MicroRNAs (miRNAs), which are small non-coding RNAs, regulate various biological processes, including innate immune responses. Toll-like receptors (TLRs) and their signaling pathways are crucial in the innate immune defense against HBV. Jiang *et al.* found that in HBV-infected HepG2.2.15 cells, there were seven upregulated and eight downregulated miRNAs associated with the TLR pathway, such as miR-194 and let-7e-5p^40^. During the progression of HBV infection, these miRNAs can modulate the immune responses of innate immune cells, including natural killer cells (NK cells), neutrophils, and dendritic cells. Therefore, regulating the expression of relevant miRNAs may emerge as a significant therapeutic strategy for liver diseases associated with HBV infection. NK cells play a role in killing during acute HBV infection, CHB and hepatitis B virus-related acute-on-chronic liver failure (HBV-ACLF), but these disease can cause a reduction in the number of NK cells and impair their function^41^-^43^. Regrettably, there are currently no well-established mouse models for HBV-ALF or HBV-ACLF. LPS-induced ALF mice exhibit neutrophil infiltration and significant formation of neutrophil extracellular traps (NETs), exacerbating liver oxidative stress and inflammatory damage^44^-^46^. Neutrophil recruitment can also be found in APAP-induced ALF mice, with neutrophil depletion being beneficial for reducing liver cell damage^47^-^49^. The role of NK cells and neutrophils in patients with HBV-ALF is unclear. In our study, the proportions of activated NK cells and neutrophils decreased in the livers of the patients in the HBV-ALF group, suggesting that these cells may play a compensatory role or be rapidly consumed. Correlation analysis revealed that SLC7A11 and HMOX1 were positively correlated with plasma cells, both of which were significantly increased in the case group, a finding that was consistent with the trend of changes in plasma cells.

Therapeutic agents targeting the hub genes were retrieved from both the DGIdb and DrugBank databases. Riluzole, an SLC7A11-targeted drug identified in both datasets, is a glutamate antagonist that has been used to treat amyotrophic lateral sclerosis^25^, ^26^. Riluzole may exert neuroprotective effects in HBV-ALF by inhibiting the excessive release of glutamate. In APAP-induced ALF, acetylcysteine can exert antioxidant effects by binding to the toxic metabolite N-acetyl-p-benzoquinone imine (NAPQI) or by providing cysteine; its antioxidant role has been more extensively studied in respiratory diseases^50^. The other six targeted drugs are related primarily to amino acid or lipid metabolism. The approved HMOX1-targeted drugs are NADH and vitamin E. NADH is involved mainly in the body's energy metabolism, while vitamin E can inhibit lipid peroxidation and oxidative stress. Acetylcysteine and Vitamin E may mitigate hepatocyte injury through their antioxidant and anti-inflammatory effects^51^. NADH may facilitate the repair and regeneration of damaged hepatocytes by enhancing energy metabolism. These findings provide a reference for identifying potential new therapies for ferroptosis in patients with HBV-ALF.

However, our study has several limitations. The samples of the GSE14668 and GSE96851 datasets are small, which may lead to bias in the final gene validation results. Additionally, there is a lack of validation against external datasets and clinical trials. Subsequently, it is essential to expand the sample size by searching additional databases or incorporating clinical patient data. Following this, an appropriate murine model should be identified to conduct preliminary functional validation of the hub genes through both *in vivo* and *in vitro* experiments.

In summary, we explored the gene expression profiles of patients with HBV-ALF and identified 55 ferroptosis-related DEGs and 45 autophagy-related DEGs. Moreover, we determined the biological processes and pathways in which these genes participate. We also respectively identified 8 and 6 key DEGs associated with ferroptosis and autophagy between two groups. These genes are interconnected through HMOX1 and are involved primarily in cellular catabolism and energy metabolism. Targeting these genes and pathways in combination with pharmacological inhibitors of ferroptosis may be a potential therapy for HBV-ALF. Through subsequent research, we can explore the association of hub genes with HBV-ALF, their mechanisms of action, and whether they have a role in modulating the immune microenvironment. This will further our understanding of the pathogenesis of HBV-ALF and potentially enhance therapeutic efficacy.

## Supplementary Material

Supplementary figures and table legends.

Supplementary table 1.

Supplementary table 2.

## Figures and Tables

**Table 1:** Different functions of ferroptosis-related DEGs (FRGs)

Driver	Suppressor	Marker	Unclassified
ABCC1, ACADSB, ACSL4, ALOX5, CDCA3, CDO1, G6PD, GLS2, GOT1, GSTZ1, HMOX1, LGMN, LPCAT3, MEG3, PGRMC1, SLC38A1, SLC39A14, SLC7A11, TF, TFR2, TIMP1, TTPA	AR, CD44, CP, DHODH, ENO3, FABP4, FANCD2, G6PD, GCH1, GCLC, GOT1, HELLS, HMOX1, KIF20A, MGST1, MPC1, MT1G, NQO1, PPARA, RARRES2, RRM2, SLC7A11, TF, TMSB4X	TF	ASNS, AURKA, BNIP3, CAPG, CXCL2, GPT2, HAMP, HMOX1, NCF2, PSAT1, RGS4, RRM2, SLC2A3, SLC7A11, STEAP3, TF, TUBE1, VEGFA

**Table 2:** Approved drugs targeting ferroptosis-related hub genes in DrugBank

Gene	Approved Drugs
SLC7A11	Riluzole (DB00740) (inducer), Acetylcysteine (DB06151) (activator), Cystine (DB00138), Glutamic acid (DB00142), Taurocholic acid (DB04348), Rosuvastatin (DB01098), Tauroursodeoxycholic acid (DB08834) (substrate), Thimerosal (DB11590) (antagonist)
HMOX1	NADH (DB00157), Vitamin E (DB00163) (inducer)
G6PD	Glycolic acid (DB03085) (inhibitor), Artenimol (DB11638) (ligand), Prasterone (DB01708) (inhibitor)
RRM2	Cladribine (DB00242) (inhibitor), Gallium nitrate (DB05260) (inhibitor)
KIF20A	/
HELLS	/
GPT2	Pyridoxal phosphate (DB00114) (cofactor), Glutamic acid (DB00142) (substrate/product of), Phenelzine (DB00780)
GLS2	/

**Table 3:** Approved drugs targeting autophagy-related hub genes in DrugBank

Gene	Approved Drugs
HMOX1	NADH (DB00157), Vitamin E (DB00163) (inducer)
SPP1	/
CCR2	/
DCN	/
IRS1	/
IGF1	/
