# Supplementary material for: Potential Biomarkers and Therapeutic Targets in Hepatitis B Virus-related Acute Liver Failure: Interplay of the Ferroptosis, Autophagy and Immune Responses
Source: Int J Med Sci. 2025 Jan 21;22(4):806–18. doi: 10.7150/ijms.106360 (PMC11843133; doi:10.7150/ijms.106360)

Supplementary legends

Table S1 The proportions of 22 types of immune cells in GSE14668

Table S2 The proportions of 22 types of immune cells in GSE96851

Figure S1 Common ferroptosis-related hub genes in the MCC and LASSO results

The R software packages "VennDiagram" were used to plot Venn diagrams. (A) After eliminating duplicate values, the least absolute shrinkage and selection operator(LASSO) identified 36 differentially expressed genes (DEGs) (B) Venn diagram of the common ferroptosis-related DEGs(FRGs) in the maximum clique centrality(MCC) and LASSO results

Figure S2 The common autophagy-related hub genes in the MCC and LASSO results

The R software packages ″VennDiagram″ were used to plot Venn diagrams . (A) After eliminating duplicate values, the least absolute shrinkage and selection operator(LASSO) identified 7 differentially expressed genes (DEGs) (B) Venn diagram of the common autophagy-related DEGs(ARGs) in the maximum clique centrality(MCC) and LASSO results.

A

```
Lasso linear model                         No. of obs         =         62
                                           No. of covariates  =         55
Selection: Cross-validation                No. of CV folds    =         10
```

ID	Description	lambda	No. of nonzero coef.	Out-of-sample R-squared	CV mean prediction error
1	first lambda	.465466	0	-0.0395	.2501341
80	lambda before	.0002992	38	0.9909	.0021824
* 81	selected lambda	.0002726	39	0.9909	.0021805
82	lambda after	.0002484	39	0.9909	.0021901
87	last lambda	.000156	40	0.9905	.0022954

* lambda selected by cross-validation.

B

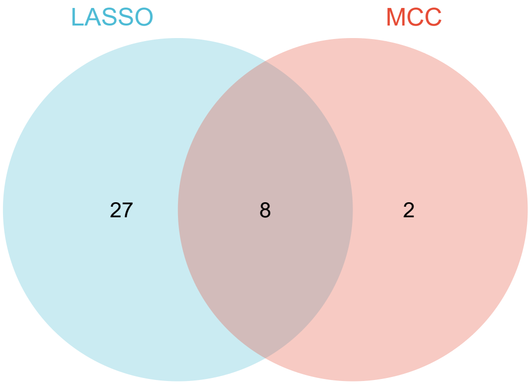

A

ID	Description	lambda	No. of nonzero coef.	Out-of-sample R-squared	CV mean prediction error
1	first lambda	.4714327	0	-0.0166	.2446243
34	lambda before	.021882	7	0.9525	.0114211
* 35	selected lambda	.019938	7	0.9526	.0114151
36	lambda after	.0181668	8	0.9526	.0114171
40	last lambda	.0125217	11	0.9521	.0115325

* lambda selected by cross-validation.

B

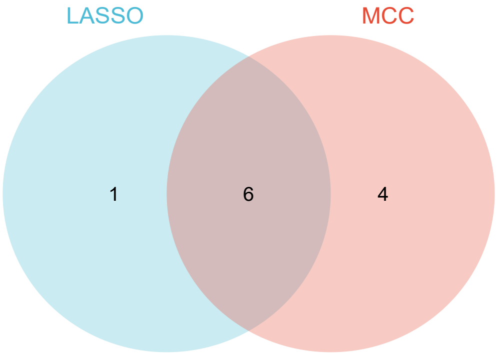

Supplement: Supplementary file 1 — Supplementary figures and table legends. [file ijmsv22p0806s1.pdf]
